# Epidemiological and Clinical Features of *Plasmodium falciparum* Malaria in United Nations Personnel in Western Bahr el Ghazal State, South Sudan

**DOI:** 10.1371/journal.pone.0055220

**Published:** 2013-01-25

**Authors:** Dengming He, Yuqi Zhang, Xiaofeng Liu, Shimin Guo, Donghong Zhao, Yunjie Zhu, Huaidong Li, Li Kong

**Affiliations:** 1 Department of Infectious Diseases, The 88th Hospital of Chinese PLA, Tai’an, Shandong Province, China; 2 Department of Gastroenterology Diseases, General Hospital of Ji’nan Military Command, Ji’nan, Shandong Province, China; 3 Department of Inf1ectious Diseases, The 150th Hospital of Chinese PLA, Luoyang, Henan Province, China; 4 The Level 2 Hospital of the United Nations Mission in the Sudan (UNMIS), Wau City, Western Bahr el Ghazal State, South Sudan; The Queensland Institute of Medical Research, Australia

## Abstract

Western Bahr el Ghazal State is located in northwestern South Sudan, which is a tropical area subject to *Plasmodium falciparum* malaria epidemics. The aim of this study is to explore the epidemiological and clinical features of *Plasmodium falciparum* malaria in United Nations personnel stationed in this area. From July 2006 to June 2009, epidemiological data and medical records of 678 patients with *Plasmodium falciparum* malaria at the U.N. level 2 hospital were analyzed. The U.N. personnel were divided into individuals not immune to *Plasmodium falciparum* and individuals semi-immune to *Plasmodium falciparum*. The patients were divided into a chemoprophylaxis group (non-immune individuals who complied with the chemoprophylaxis regimen, 582 cases) and a no/incomplete chemoprophylaxis group (non-immune individuals who either did not fully comply with chemoprophylaxis or did not use it at all and semi-immune individuals who did not use chemoprophylaxis, 96 cases). Overall morbidity was about 11.3%. There was a significant difference in the morbidity of semi-immune and non-immune individuals (1.3% vs. 15.1%, *P*<0.001). Out of the total, 82.9% of cases occurred during the rainy season. The incidence of fever in the chemoprophylaxis group was significantly lower than in the no/incomplete chemoprophylaxis group (36.8% vs. 96.9%, *P*<0.001). Significant differences were observed between the two groups with respect to all other malaria-like symptoms except gastrointestinal symptoms, serum glucose level, platelet count, and alanine aminotransferase level. The incidence of complications was 1.2% (chemoprophylaxis group) and 44.8% (no/incomplete chemoprophylaxis group).The most common complication was thrombocytopenia, which was seen in 40.6% of the no/incomplete chemoprophylaxis group. In summary, *Plasmodium falciparum* malaria mainly occurred in rainy season. Gastrointestinal symptoms are an important precursor of malaria. Blood smears and rapid diagnostic tests should be performed after the onset of gastrointestinal symptoms. Appropriate chemoprophylaxis is necessary for reducing the severity of malaria.

## Introduction

Malaria is considered a significant threat to the health and effectiveness of military forces deployed in malaria-endemic areas. [1], [2] Western Bahr el Ghazal State is located in northwestern South Sudan, which is a tropical area subject to *Plasmodium falciparum* malaria epidemics. [3] Wau city (7°42′N, 28°0′E) is the state capital. The climate is tropical with two seasons, dry from November to March and rainy the rest of the year. The average temperature ranges between 21 and 35°C, and average relative humidity is 52.8%.

According to resolution 1590 of the Security Council (2005), the United Nations Mission in the Sudan (UNMIS) was established to advance the peace process in Sudan. About 2,000 personnel of the United Nations (U.N.) have been deployed to Western Bahr el Ghazal State since 2006, and they are alternated yearly. When South Sudan became an independent country in 2011, the Security Council established the United Nations Mission in the Republic of South Sudan (UNMISS). At present, there are 8,000 U.N. personnel in South Sudan.

There have been almost no reports on *Plasmodium falciparum* malaria in Western Bahr el Ghazal State. In this report, we summarized the epidemiology, clinical symptoms, laboratory indices, antimalarial treatment, complications, and outcome features of cases of *Plasmodium falciparum* malaria among U.N. personnel in this area. These results may be useful in the prevention and treatment of *Plasmodium falciparum* malaria among U.N. personnel and travelers in South Sudan.

## Results

### Demography and Behavior Characteristics

From July 2006 to June 2009, 678 cases (665 men and 13 women) of *Plasmodium falciparum* malaria in U.N. personnel were diagnosed by light microscopy of thick and thin stained blood smears and/or rapid diagnostic tests (RDTs) at the level 2 hospital of UNMIS in Wau. The mean age was 33.9 years (range: 21–52). In the behavioral questionnaire, all individuals claimed to have complied with regulations regarding the use of mosquito nets. However, 1.4% of respondents admitted to not having complied with the use of repellents and 14.2% of the cases had not complied with the chemoprophylaxis regimen.

### Epidemiological Features

**Figure 1:**
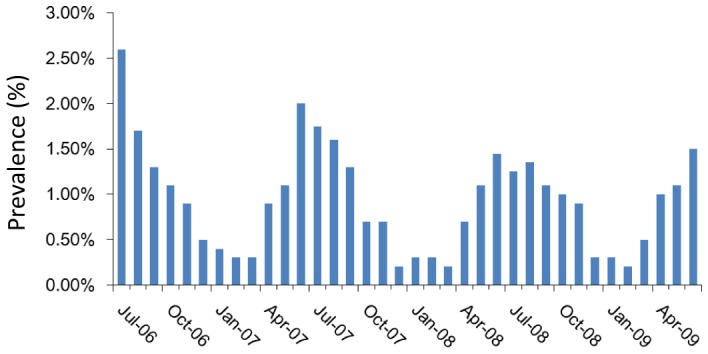
Temporal distribution of prevalence of *Plasmodium falciparum* malaria based on the U.N. populations at the UNMIS level 2 hospital in Wau, July 2006–June 2009.

The overall morbidity was about 11.3% (678/6,000). There was a significant difference in the morbidity of semi-immune and non-immune individuals: about 1.3% (23/1,800) vs. about 15.1% (635/4,200) (*P*<0.001). There were about 582 cases in the chemoprophylaxis group and 96 in the no/incomplete group, to a total of 678. In the no/incomplete chemoprophylaxis group, only 23 (23.9%) cases admitted to taking no chemoprophylaxis. Prevalence over time is shown in Figure 1. There were 562 (82.9%) cases during the rainy season and 116 (17.1%) cases during the dry season. Similar patterns associated with season were observed across the study period with peaks from June through August and the lowest morbidity from December to January.

### Signs, Symptoms, and Complications

The clinical signs and symptoms of the two groups are shown in Table 1. Fever was the most common symptom in the no/incomplete chemoprophylaxis group but not in the chemoprophylaxis group (96.9% vs. 36.8%, OR = 0.019, *P*<0.001). There were no significant difference between the two groups with respect to the proportion of any gastrointestinal symptoms except vomiting: anorexia (81.1% vs. 84.4%, OR = 0.795, *P* = 0.443), abdominal pain (46.7% vs. 53.1%, OR = 0.774, *p* = 0.245), diarrhea (50.5% vs. 43.8%, OR = 1.313, *P* = 0.219), nausea (47.4% vs. 49.0%, OR = 0.940, *P* = 0.780), and vomiting (18.6% vs. 30.2%, OR = 0.526, *P* = 0.008). However, significant differences were observed in other signs (hyperpyrexia, lung findings, hepatomegaly, splenomegly, and neck stiffness, etc.; *P*<0.001) and symptoms (chills, diaphoresis, dizziness, headache, myalgias, and dry cough, etc.; *P*<0.001).

**Table 1:** Signs and symptoms among cases of *Plasmodium falciparum* malaria between the two groups.

	Chemoprophylaxis group % (n = 582)	No/incomplete chemoprophylaxis group % (n = 96)	OR	95%CI	*P* value
Symptoms					
Fever #	36.8	96.9	0.019	0.006–0.060	<0.001
Chills	30.6	95.8	0.019	0.007–0.053	<0.001
Diaphoresis	33.3	94.8	0.027	0.011–0.069	<0.001
Dizziness	52.4	86.5	0.172	0.094–0.316	<0.001
Headache	35.9	79.7	0.003	0.000–0.047	<0.001
Myalgia	31.4	74.0	0.161	0.099–0.263	<0.001
Nausea	47.4	49.0	0.940	0.610–1.448	0.780
Vomiting	18.6	30.2	0.526	0.325–0.853	0.008
Abdominal pain	46.7	53.1	0.774	0.502–1.193	0.245
Diarrhea	50.5	43.8	1.313	0.850–2.027	0.219
Anorexia	81.1	84.4	0.795	0.441–1.432	0.443
Dry cough	7.9	29.2	0.208	0.122–0.355	<0.001
Signs					
Hyperpyrexia*	0	15.6	0.005	0.000–0.076	<0.001
Lung findings	0.7	19.8	0.028	0.009–0.085	<0.001
Hepatomegaly	0	3.1	0.023	0.001–0.448	<0.001
Splenomegly	0	4.2	0.018	0.001–0.330	<0.001
Neck stiffness	0	6.3	0.012	0.001–0.214	<0.001

# Temperature under the arm >37.5°C.

* Core body temperature >40°C.

The distribution of complications in the two groups is shown in Table 2. Only 1.2% (7/582) of the cases in the chemoprophylaxis group developed complications, but 44.8% (43/96) of the cases in the no/incomplete chemoprophylaxis group did. There was a significant difference in the incidence of complications between the two groups (*P*<0.001). The most common complication was thrombocytopenia. In the no/incomplete chemoprophylaxis group, the rates of thrombocytopenia, anemia, jaundice, hypoglycemia, hypopotassamia, hemoglobinuria, and pneumonia were 40.6%, 9.3%, 3.1%, 7.3%, 17.7%, 1.0%, and 2.1%, respectively. In chemoprophylaxis group, these rates were only 0.9%, 0.3%, 0%, 0.2%, 0.3%, 0%, and 0%, respectively.

**Table 2:** Distribution of complications among the cases in the two groups.

Complications	No/incomplete chemoprophylaxis group		Chemoprophylaxis group	
	No. (%)	Mean (range)	No. (%)	Mean (range)
Present	43 (44.8)		7 (1.2)	
Thrombocytopaenia^1^ (×10^9^/L)	39 (40.6)	78 (68–87)	5 (0.9)	83 (77–89)
Anemia^2^ (g/L)	9 (9.3)	105 (86–111)	2 (0.3)	114, 109
Jaundice^3^ (µmol/L)	3 (3.1)	55 (45–66)	0 (0)	NA
Hypoglycemia^4^ (mmol/L)	7 (7.3)	2.6 (2.3–2.8)	1 (0.2)	2.8
Hypopotassaemia^5^ (mmol/L))	17 (17.7)	3.1 (2.7–3.4)	2 (0.3)	3.3, 3.2
Hemoglobinuria	1 (1.0)	NA	0 (0)	NA
Pneumonia	2 (2.1)	NA	0 (0)	NA

1 Platelet <100×10^9^/L;

2 Hemoglobin <120 g/L;

3 Total bilirubin >43 µmol/L;

4 Plasma glucose <2.8 mmol/L;

5 Plasma potassium <3.5 mmol/L.

### Laboratory Indices

Considering that 44.8% of the no/chemoprophylaxis cases were cases with complications, so the group was divided into uncomplicated group and complications group. Laboratory indices are shown in Table 3. There were significant differences in serum glucose level, hemoglobin, platelet count, alanine aminotransferase (ALT) level, and parasite density among three groups. However, no significant differences were seen regarding white blood cell count. There were significant differences in serum glucose level, hemoglobin, platelet count, and parasite densities between cases with complications and both the chemoprophylaxis group and the uncomplicated cases.

**Table 3:** Laboratory indices of cases among chemoprophylaxis group, uncomplicated group, and complications group (mean±SD).

Laboratory indices	Chemoprophylaxis group (n = 582)	No/incomplete chemoprophylaxis group		*P* value
		Uncomplicated (n = 53)	Complications(n = 43)	
White blood cell (×10^9^/L)	6.3±1.5	6.1±1.4	6.3±1.2	0.377
Serum glucose (mmol/L)	5.1±0.6	4.6±0.6	4.1±0.9	<0.001*
Hemoglobin (g/L)	145±10	141±9	128±18	<0.001#
Platelet count (×10^9^/L)	164±28	166±43	82±18	<0.001#
ALT (IU/L)	36±7	42±10	44±9	<0.001^^^
Parasite densities (parasites/µl)	130±70	480±200	710±280	<0.001*

* Significant differences were observed between any two groups.

# Significant differences were observed between the chemoprophylaxis group and complications group or between the uncomplicated group and complications group.

^Significant differences were observed between chemoprophylaxis group and uncomplicated group or between the chemoprophylaxis group and complications group.

### Treatments and Outcomes

Patients with uncomplicated *Plasmodium falciparum* malaria were treated with an artemisinin-based combination therapy (ACT) according to World Health Organization (WHO) guidelines, mainly dihydroartemisinin-piperaquine (92.1%). Cases with complications were admitted to our hospital and treated with artesunate followed by a complete course of dihydroartemisinin-piperaquine as soon as each patient was able to take oral medicines. Other antimalarial medicines included quinine, sulfadoxine-pyrimethamine, and mefloquine.

The outcome of the regimen was satisfactory. One patient died of severe cerebral malaria, but all other patients were cured, regardless of whether they had complications. The patient who died was 48 years old, semi-immune to malaria, and had undergone no chemoprophylaxis. The questionnaire indicated no special circumstances. Among the other cases, the parasites were eliminated in 100% of cases who underwent treatment lasting seven to ten days as assessed using light microscopy of thick and thin stained blood smears.

## Discussion

*Plasmodium falciparum* is responsible for over 97% of malarial infections in Sudan. [3] Western Bahr el Ghazal State is located in the northwestern part of South Sudan, whose temperature and humidity are suitable for reproduction of *Plasmodium falciparum*, especially during the rainy season. The temporal distribution characterized by onset during the rainy season observed in the cases evaluated in this study is consistent with the epidemiological features of malaria in Sudan.

*Plasmodium falciparum* is the dominant species of parasite in Sudan, where attempts to reduce the number of cases have had limited success. [3] The UNMIS population in Western Bahr el Ghazal State came mainly from China, India, Pakistan, Bangladesh, Kenya, and some countries in Europe and North and South America. According to the WHO, the proportion of cases attributable to *Plasmodium falciparum* in China, India, and Pakistan is 6%, 55%, and 31%, respectively. [4] Among these countries, Kenya is the only country considered a *Plasmodium falciparum* malaria epidemic area. The UNMIS population evaluated in this area can be divided into people who are not immune and people who are semi-immune to *Plasmodium falciparum* malaria. Non-immunes face a higher risk of *Plasmodium falciparum* malaria infection than local residents and semi-immunes. Chemoprophylaxis and mosquito-fighting measures are mandatory for non-immunes in UNMIS. Because of its long half-life, mefloquine is the preferred chemoprophylactic agent against *Plasmodium falciparum* malaria in UNMIS. However, its side effects, which include vomiting, dizziness, syncope, extrasystoles, tinnitus, and emotional problems, cause some people refuse or cease mefloquine.

There is a significant difference in overall morbidity between non-immunes and semi-immunes. Even among non-immune individuals given mefloquine as part of a chemoprophylaxis regimen, high morbidity (15.1%) is associated with susceptibility to *Plasmodium falciparum.* This indicates that chemoprophylaxis should not be expected to prevent *Plasmodium falciparum* infection in non-immunes. In view of the side effects of mefloquine, UNMIS does not recommend high mefloquine dosage or combination with other drugs for the purpose of prevention. However, even semi-immune individuals who do not undergo chemoprophylaxis, low morbidity (1.3%) is with immunity to *Plasmodium falciparum* and with age.

The symptoms of malaria include paroxysms defined by intense chills, fever, and sweating caused as new merozoites burst from the erythrocytes and infect more cells. [5] In general, the majority of patients experience fever (>92%), chills (79%), headaches (70%), and diaphoresis (64%). [6] In the chemoprophylaxis group, a large proportion of 63.2% afebrile *Plasmodium falciparum* infections were associated with regular chemoprophylaxis. In the no/incomplete chemoprophylaxis group, fever (96.9%), chills (95.8%), and headache (79.7%) were typically seen. Significant differences were also observed in all other signs and symptoms except gastrointestinal symptoms. Although it does not completely prevent parasite infection, standard chemoprophylaxis still plays a crucial role in reducing the symptoms and prevalence of severe malaria. For this reason, many patients taking chemoprophylaxis present with atypical malaria symptoms. This suggests that malaria tests in hospitals in malaria endemic countries must be a routine clinical management, especially for new staff and travelers from other areas.

However, most gastrointestinal symptoms, specifically anorexia, diarrhea, nausea, and abdominal pain, showed no significant difference between the two groups. Incidences of all symptoms were significantly higher than in previous reports. [7] This showed gastrointestinal symptoms to be dominant features of *Plasmodium falciparum* malaria in Western Bahr el Ghazal State, even with appropriate chemoprophylaxis. For this reason, malaria should be considered and tested when any new patient reports gastrointestinal symptoms in Western Bahr el Ghazal State.

Complications were observed primarily in the no chemoprophylaxis group. In one study of travelers returning from the tropics, thrombocytopenia and hyperbilirubinemia had a positive predictive value of 95% for malaria. [8] In this study, thrombocytopenia was found to be the most common complication, followed by hypopotassemia and anemia. This was consistent with previous studies. One previous study showed thrombocytopenia to be the most common laboratory abnormality (60% of cases), followed by hyperbilirubinemia (40%), anemia (30%), and elevated aminotransferase levels (25%). [9] Regarding laboratory indices, significant differences in parasite densities were observed between categories. Parasite density was associated with changes in laboratory indices and in complication rates. Significant differences in serum glucose level and platelet count were observed between cases with complications and uncomplicated cases.

According to WHO guidelines, artemisinin derivatives should not be used as monotherapies for the treatment of uncomplicated malaria because this can promote resistance to this critically important class of antimalarial agents. [10] ACTs are the recommended treatment for uncomplicated *Plasmodium falciparum* malaria. [10] The ACTs recommended by WHO include artemether-lumefantrine, artesunate-amodiaquine, artesunate-mefloquine, dihydroartemisinin-piperaquine, and artesunate-sulfadoxine-pyrimethamine. [10] Regarding other medicines, an in vivo study in rural eastern Sudan showed sulfadoxine-pyremethamine or sulfadoxine-pyremethamine+chloroquine combination treatment alone cured fewer than 70% of patients with uncomplicated *Plasmodium falciparum* malaria. [11].

In this study, dihydroartemisinin-piperaquine-based treatment showed satisfactory effects including a high rate of parasite clearance after a standard course of treatment. In a randomized single-blinded clinical trial, dihydroartemisinin-piperaquine was found to be superior to artemether-lumefantrine for reducing the risk of recurrent parasitemia and gametocytemia. It also fostered better hemoglobin recovery in the treatment of uncomplicated *Plasmodium falciparum* malaria. [12].

However, a few limitations should be considered in this study. Microscopy is the preferred diagnostic test for patients with severe febrile illness. Because sensitivity of RDTs is low when the parasite density <100/µl, the diagnosis of afebrile or atypical malaria with low parasitemia mainly depended on microscopy. [13], [14] Therefore, the quality of microscopists may bring the possibility of high false positive rates in the microscopy test, especially in patients with low parasite density.

In conclusion, *Plasmodium falciparum* malaria has a high morbidity among U.N. personnel in Western Bahr el Ghazal State, especially during the rainy season. Patients should be evaluated for malaria using blood smears and RDTs at the onset of gastrointestinal symptoms. Dihydroartemisinin-piperaquine is an effective medicine for treatment. Appropriate chemoprophylaxis is necessary to reduce the severity of malaria.

## Materials and Methods

### Ethics

The analysis was conducted on anonymized data, collected as part of routine patient care. No additional investigations were performed. Therefore, no prior informed consent from the patients was required. The study protocol conformed to the ethical guidelines of the 1975 Declaration of Helsinki as reflected in *a priori* approval by the ethics committee of the level 2 hospital of UNMIS in Wau and the 88^th^ Hospital of the People’s Liberation Army. Our ethics committee waived the need for informed consent.

### Study Setting

The UNMIS level 2 hospital in Wau is responsible for the health of about 2,000 people deployed in the Western Bahr el Ghazal State. The average rotation period of the population is one year. These people come from over twenty countries, including China, India, Pakistan, Kenya, Bangladesh, and some countries in Europe and North and South America. To explore the epidemiological and clinical features of *Plasmodium falciparum* malaria among U.N. employees and representatives in Western Bahr el Ghazal State, 678 cases of *Plasmodium falciparum* from July 2006 to June 2009 were retrospectively reviewed in this study.

### Diagnosis and Subjects

As indicated by the high prevalence of *Plasmodium falciparu*m malaria in Western Bahr el Ghazal State, all patients who arrived at the hospital were routinely tested for *Plasmodium falciparum*. *Plasmodium falciparum* malaria was diagnosed by light microscopy of thick and thin stained blood smears and/or rapid diagnostic tests (RDTs). The diagnosis of 41 case (5.9%) with typical febrile malaria and 188 cases (27.4%) with afebrile or atypical malaria mainly depended on microscopy. The combination test of microscopy and RDTs were used in diagnosis of the other 449 cases. The proportion diagnosed by microscopy and RDTs in this later group was 96.7% and 98.0%, respectively. Parasitemia was determined using thick films by counting the number of parasites per 200 leucocytes and assuming that each subject had 8000 leucocytes per µl. The thin smears were used to identify malaria parasite species.

To obtain the information on behavioral factors an anonymous epidemiological study with behavioral questionnaire was conducted among the cases. Oral informed consent was obtained in advance from all participants. Once diagnosed with *Plasmodium falciparu*m malaria, the patients were asked to complete a behavioral questionnaire covering such topics as the use of mosquito nets, chemoprophylaxis, long pants, long sleeves, and repellents. Patients co-infected with other pathogens, which can cause malaria-like symptoms were excluded from the study. In non-immune individuals, the half-life of mefloquine and the incubation phase of *Plasmodium falciparu*m malaria were used to identify people who had not complied with chemoprophylaxis. Patients whose treatment had lapsed for two to four weeks were considered to have undergone incomplete chemoprophylaxis. Cases whose treatment had lapsed for over four weeks were considered to have had no chemoprophylaxis.

### Groups

The U.N. population was divided into a semi-immune group (e.g. Kenyan contingent, about 600 people per year) and non-immune group (e.g. European, Chinese, Indian, and Pakistani contingents; about 1,400 people per year), according to the *Plasmodium falciparum* malaria endemic areas. In view of the difference in chemoprophylaxis in the questionnaire, the patient population was differentiated into those who complied with chemoprophylaxis (chemoprophylaxis group) and those who did not use chemoprophylaxis or did not comply with chemoprophylaxis (no/incomplete chemoprophylaxis group). The former group contained only non-immune individuals. The latter included non-immunes who did not use or comply with chemoprophylaxis because of side effects. It also included semi-immunes who did not use chemoprophylaxis. According to WHO recommendations, mefloquine at a dose of 250 mg per week was the preferred chemoprophylaxis in UNMIS. Although compulsive for non-immunes, chemoprophylaxis is not recommended for semi-immunes.

### Data Analysis

Clinical data were analyzed using SPSS version 18 software. Comparisons were made between two groups in terms of observable symptoms, physical findings, laboratory indices, and complications. All tests for significance and resulting *P* values were two-sided, with significance cutoff of 0.05. Proportions were compared using the Chi-square test and means were compared using the Student’s t-test and ANOVA.
